# PSON: A Serialization Format for IoT Sensor Networks

**DOI:** 10.3390/s21134559

**Published:** 2021-07-02

**Authors:** Álvaro Luis, Pablo Casares, Juan J. Cuadrado-Gallego, Miguel A. Patricio

**Affiliations:** 1 Internet of Thinger, Thinger.io, Madrid, Spain; alvarolb@thinger.io; 2 Ultra Tendency International GmbH, 39326 Colbitz, Germany; pablo.casares@ultratendency.com; 3 Department of Computer Science, Universidad de Alcalá, Alcalá de Henares, 28805 Madrid, Spain; jjcg@uah.es; 4 Department of Computer Science and Software Engineering, Concordia University, Montreal, QC H3G 1M8, Canada; 5 Applied Artificial Intelligence Group, Universidad Carlos III de Madrid, Colmenarejo, 28270 Madrid, Spain

**Keywords:** data processing, data serialization, internet of things

## Abstract

In many Internet of Things (IoT) environments, the lifetime of a sensor is linked to its power supply. Sensor devices capture external information and transmit it. They also receive messages with control commands, which means that one of the largest computational overheads of sensor devices is spent on data serialization and deserialization tasks, as well as data transmission. The simpler the serialization/deserialization and the smaller the size of the information to be transmitted, the longer the lifetime of the sensor device and, consequently, the longer the service life. This paper presents a new serialization format (PSON) for these environments, which simplifies the serialization/deserialization tasks and minimizes the messages to be sent/received. The paper presents evaluation results with the most popular serialization formats, demonstrating the improvement obtained with the new PSON format.

## 1. Introduction

The next generation of telecommunications networks (fifth generation or 5G) aims to redefine the rules of the game in connectivity in many respects. The capabilities of the new generation are overwhelming: data rates of up to 10 Gbps (10–100 times better than the current 4 and 4.5G networks), latencies of 1 millisecond, network availability of 99.999%, 100% coverage, 90% reduction in network power consumption, and increased capacity of simultaneously connected users are expected [1]. Beyond improved speed, or latency, 5G is expected to unleash a massive IoT (Internet of Things) ecosystem [2] in which networks can meet the communication needs of billions of connected devices with the right capabilities [3].

The 5G specifications and use cases go far beyond mobile communication, the service experienced by the end user. Specifically, the new 5G standard defines different operational scopes or categories, which are eMBB (enhanced Mobile Broadband), URLLC (Ultra-Reliable Low-Latency Communications), and mMTC (massive Machine Type Communications) [4].

The aim of eMBB is to substantially improve the bandwidth of mobile communications with moderate latency and thus provide a solution for emerging applications related to virtual reality, augmented reality, UltraHD quality applications, 360° video streaming, etc., which will be further enhanced in the future; for example, it will be boosted in 2021.

On the other hand, URLLC [5] refers to ultra-reliable communications with very low communication latency. URLLC will support a range of advanced services for latency-sensitive connected devices to enable applications across a wide spectrum, such as factory automation, autonomous driving, industrial internet, remote surgery, and smart grids, among others.

In addition, within the proposed 5G specification, mMTC services [4], which are massive machine-to-machine communications, are also defined. These are specifications focused on providing a cost-effective and robust connection to billions of devices without overloading the network. It will serve devices whose typical use case is to send small amounts of information on a regular basis, enabling optimal use of the power of IoT devices, as the vast majority of IoT devices are battery-powered.

Within the Internet of Things, 5G’s mMTC technology is being postulated as an unprecedented connectivity solution for the development of connected products and services [6,7]. The specification sets requirements around networking devices with up to 10 years of battery life, coverage penetration of 164 dB with a capacity of 160 bits per second, coverage density to support up to one million connected devices per square kilometer, communication latencies of less than 10 s for 20 bytes of data, and a crucial element for massive scaling: very inexpensive hardware [4]. In addition, this specification states that 5G networks and mMTC should support more features and applications over time, such as positioning, mobility, or multicast communication capability, among others. The mMTC will rely on two network standards, NB-IoT (Narrowband-IoT) and LTE-M, which are 3GPP specifications particular to IoT [8].

NB-IoT and LTE-M are part of LPWANs (Low-Power Wide-Area Networks), such as Sigfox, ZigBee, and LoRa [9], which are technologies that offer ranges of several kilometers and low power consumption. They mitigate the shortcomings of WPANs (Wireless Personal Area Networks), such as WiFi and Bluetooth, which, although still widespread [10], have limitations with respect to their range (only a few tens of meters) and their power consumption [11], which is why they cannot be extensively used in IoT contexts. The main advantage of the LPWAN approach in 5G compared to other technologies is that they are within the licensed spectrum (so they are immune to interference) and use the infrastructure of telephony networks, so there is no need to deploy their own infrastructure. Moreover, because it is a standardized technology, LPWAN is supported by a global ecosystem that allows interoperability between different market players and production scales of these solutions, which will reduce the cost of the technology once it is consolidated [12].

As discussed above, the massive growth of IoT solutions will require the use of devices with limited capabilities [13,14]. Although technological advancement is ongoing, due to the limited resources available in a typical sensor device, and in order to achieve lower power consumption at the nodes, it is important to reduce the amount of data exchanged. Various initiatives have been developed to improve energy consumption in sensor networks. Some of them aim to optimize energy by designing routing protocols [15,16]. Other work has focused on the development of Wireless Power Transfer (WPT) technology [17,18,19,20,21] or Energy Harvesting (EH) [22]. However, the focus of this research work is on the efficient transfer of data from devices to the server-side. In this way, the processing power, available memory, and battery life of IoT devices, which are mostly limited in these aspects [23,24], will be optimized.

For the transmission of information in both directions, that is, between the device and the server, serialization formats are used. Serialization is the process of translating data structures or object states into a format that can be transmitted and reconstructed later. Therefore, serialization is the conversion of an object into a sequence of bytes, whereas deserialization is the reconstruction of an object from a sequence of bytes. Serialization/deserialization processes are critical for devices with limited on-board energy, such as those in an IoT network. The smaller the size of the serialized object and the shorter the execution time involved, the more efficient the format. Any reduction in processor time for transaction serialization/deserialization contributes to an increase in the deployed lifetime of an IoT device. There are a number of different serialization formats, as is discussed further below. In IoT environments, where many devices are expected to be connected to the server, the importance of selecting a serialization format is vital in order to reduce overheads (measured as memory and bandwidth usage) [25].

Although the selection of the message protocol [25,26] is also relevant in the communication system, the focus of this paper is the presentation of a new serialization format called PSON [27]. The main goal of PSON is to define a serialization format efficiently in terms of total serialization time and bandwidth required to transmit arbitrary data payloads. The main problem with other encoding technologies is that they were not specifically designed thinking in the IoT ecosystem, both for servers to allow them to scale better while decoding massive IoT sensor data, and for sensors to last long when powered by batteries. This way, some existing methods are quite efficient for reducing the payload size, but they increase the total serialization/deserialization complexity in terms of processing power, especially for a small microcontroller. PSON is then focused on providing a balance between serialization time and generated payload size. PSON is used in the Thinger.io Cloud Platform [28]. Thinger.io is an open-source platform with capabilities for the collection, management, and analysis of a huge amount of heterogeneous sensor data. The use of PSON provides optimization in terms of execution time, channel utilization, and power consumption compared to the most common methods used in IoT environments. The aim of this paper is to describe this new serialization format and assess its performance compared to the most widely used formats.

The remainder of this article is organized as follows: Section 2 introduces the main aspects and the information sources of the data serialization formats that are analyzed and compared in the presented research. Section 3 provides an in-depth description of the new developed data serialization format, PSON. Section 4 describes the design of the research carried out to compare the selected data serialization formats and specifically addresses the attributes used to perform the comparison, the hardware used and its relation with IoT, the libraries used, and the test and payloads used. Section 5 presents the research results obtained from each attribute analyzed and the hardware used. Finally, Section 6 summarizes the conclusions obtained in the research and describes possible future work that can be performed. Taking this research as a starting point, some of that research has already begun.

## 2. Data Serialization Formats

This paper presents a comparison between data serialization formats; in this section, we enumerate and describe the main characteristics of the data serialization formats included in the comparison. The data serialization formats included in this study are those with widespread use:

- JSON, JavaScript Object Notation. The European Computers Manufacturers Association, ECMA, published the ECMA-404 standard, “The JSON data interchange syntax”, whose latest update was the 2nd edition, published in 2017 [29]. This document presents the most recent version of the standardized JSON language. As defined in the document, “JSON is a lightweight, text-based, language-independent data interchange format. It was derived from the ECMAScript programming language, but is programming language independent. JSON defines a small set of structuring rules for the portable representation of structured data”. JSON is based on a subset of the JavaScript Programming Language Standard ECMA-262 3rd Edition published in December 1999, and it is a very stable data-interchange language that has had few modifications since it was first presented in 2001 on the JSON organization website [30]. This stability is complemented by the fact that it uses conventions similar to the C-family of languages, such as C, C++, C#, Java, JavaScript, Perl, and Python, making JSON one of the most widely used data serialization formats.
- BSON, Binary JSON. First developed by MongoBD [31] as a binary structure that encodes type and length information, BSON is currently maintained as an open binary-encoded serialization of JSON-like documents in [32], whose latest published specification version is 1.1. This document describes the three characteristics for which BSON was designed: “Lightweight, Keeping spatial overhead to a minimum; Traversable, was designed to be tranversed easily; Efficient, Encoding data to BSON and decoding from BSON can be performed very quickly in most languages due to the use of C data types.”
- Protocol Buffers, developed by Google as a mechanism for serialized structured data. Two versions have been published, Proto2 and Proto3, the specifications of which can be found in [33]. The most recent version of Protocol Buffers, proto3, supports generated code in Java, Python, Objective-C, Dart, Go, Ruby, and C#. The main objective of Protocol Buffers is to be a small, fast, and simple mechanism for data serialization and be language-neutral, platform-neutral, and extensible.
- XML, Extensible Markup Language. Developed by six different XML Groups [34], each dedicated to a different aspect of the Information and Knowledge Domain, W3C [35], Extensible Markup Language (XML) is a text format derived from SGML in ISO 8879, which was designed to meet the challenges of large-scale electronic publishing, but the file was extensively used in the exchange of a wide variety of data on the Web and elsewhere. Its first publication was in 1997, and since then, many different specifications, which can be found in [36], have been published.
- YAML, YAML Ai not Markup Language. Developed as an international collaboration, YAML resulted from the serialization format for Inline, Data::Denter module, developed by Ingy dot, and a simplification of XML, developed by Clark Evans and Oren Ben-Kiki. The first specification was published in 2001, and the current version is YAML 1.2, published in 2009. All of the specifications can be found in [37]. YAML was integrated and built upon concepts of C, Java, Perl, Python, Ruby, RFC0822 (MAIL), RFC1866 (HTML), RFC2045 (MIME), RFC2396 (URI), XML, SAX, and SOAP.
- MessagePack. Developed by Sadayuki Furuhashi in 2009, MessagePack is a binary serialization format that enables data exchange among multiple languages. Small integers are encoded into a single byte, and typical short strings require only one extra byte in addition to the strings themselves. There is only one specification, with the most recent update in 2017, which can be found in [38].
- Apache Thrift. Developed by Facebook, it was open sourced in 2007 and entered the Apache Incubator in 2008, becoming an Apache Top-Level Project (TLP) in 2010. It is rigorously maintained, and its latest release was published in March 2021. This and all previous releases since 2009 can be found in [39]. Apache Thrift allows reliable performance communication and data serialization across a variety of programming languages and use cases. The project team aimed for Thrift to embody several characteristics: Simplicity, with a simple and approachable code, free of unnecessary dependencies; Transparency, conforming to the most common idioms in all languages; Consistency, with niche, language-specific features in extensions, not the core library; and Performance, striving for performance first, elegance second.
- Apache Avro. Avro joined the Apache Software Foundation as a Hadoop subproject in 2009. Since then, it has been very intensively maintained, and more than thirty releases have been published, with the latest one being 1.10.2 in 2021. All versions can be found in [40]. This is a data serialization system that relies on schemas. When Avro data is read, the schema used when writing it is always present. This permits each datum to be written with no per-value overheads, which also allows its use with dynamic scripting languages since the data, together with their schema, are fully self-describing. The developer team indicates that Avro is intended to provide rich data structures; a compact, fast, binary data format; a container file to store persistent data; a remote procedure call (RPC); and simple integration with dynamic languages based on the fact that code generation is not required to read or write data files, nor do RPC protocols need to be implemented. For this reason, code generation is an optional optimization step and is only worth implementing for statically typed languages.

## 3. PSON: Thinger.io Data Serialization Format

PSON is an object serialization specification similar to JSON but specifically created for microcontrollers. It improves JSON in encoding/decoding complexity and generates a more compact representation over the wire. It also extends JSON by allowing any arbitrary binary information to be encoded, which is not permitted by the standard JSON schema. Thus, PSON handles different data types, which are referenced in the following as the wire type:

- Unsigned: represents unsigned integers;
- Signed: represents signed integers;
- Float: represents both IEEE 754 simple and double precision;
- Discrete: represents discrete values, such as true, false, or null;
- String: represents a UTF-8 string;
- Bytes: represents a byte array;
- Map: represents key–value pairs of objects;
- Array: represents a sequence of objects.

**Figure 1:**
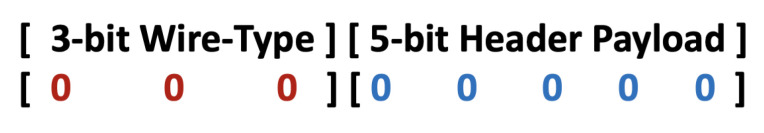
PSON data header composed of both wire type (3 bits) and header payload (5 bits).

To represent this kind of heterogeneous information, PSON messages are encoded as series of header–value pairs. Headers indicate the type of data, and the value represents the actual value. Therefore, a decoder needs to read the header to retrieve the actual data type and determine how to decode the upcoming value. A header is a fixed single byte composed of two fields: the wire type and the header payload. The wire type is encoded in the first 3 MSB (most significant bits), while the payload header is kept on the remaining 5 LSB (less significant bits). Figure 1 represents this structure.

The wire-type field of the header has a clear role in describing the value type, i.e., a number, a float, a string, an object, etc. On the other side, the header payload is 5-bit general-purpose storage that is used to optimize the serialization size. In this case, 5 bits allows up to 32 different values to be specified, which, in PSON, is used for different purposes:

- Represent small signed/unsigned integers (0–30);
- Indicate whether the floating point value is an IEEE 754 with simple or double precision;
- Discern between true, false, and nulls;
- Indicate the string size (up to 30 characters);
- Indicate the byte array size (up to 30 bytes);
- Indicate the number of elements in a map (up to 30 elements);
- Indicate the number of elements in an array (up to 30 elements).

Thus, a header contains the wire type and, under some circumstances, the actual value or size of the upcoming object/array, resulting in an efficient encoding representation. This is especially useful in the embedded ecosystem, where payloads tend to be small due to network and battery constraints. If an integer, length, size, or number of elements does not fit in the 5-bit storage (it is greater than 30), then it is flagged with a 0x1f (31) value in the header payload, and in this case, the actual value is represented by a varint number following the header.

Varints, also known as Little Endian Base 128 (LEB128) [41], allow small numbers to be stored in a single byte while also allowing the encoding of arbitrarily long numbers. Each byte in LEB128, except for the last byte, has the MSB set, and this indicates that there are further bytes to come. The lower 7 bits of each byte are used to store the two’s complement representation of the number in groups of 7 bits, starting with the least significant group.

**Figure 2:**
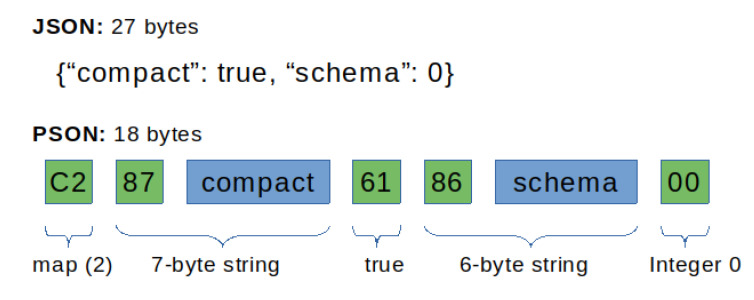
PSON encoding example versus default JSON.

The conventions used for encoding all different types are summarized in Table 1, along with their binary representations. Figure 2 presents an example of the complete encoding of a JSON document to the PSON format. In this example, a map is encoded with two keys. A map wire-type is encoded by convention with a header starting with 0b110…… The remaining bits are used for the header payload, which contains the number of elements in the map. Thus, the header value is encoded as 0b11000010, which is 0xC2, as shown in the figure. The map header is followed by key–value pairs composed of a key as the string and a value that can be any other value, such as an integer, boolean, another string, a null, etc. In the present example, it is a header encoded with 0x87, meaning that it is a string with a length of 7 bytes, as 0b100…… represents a string wire-type. Thus, the following 7 bytes contain the actual string, which is “compact”. In the following, the actual value is encoded as 0x61. In Table 1, a discrete value is encoded as 0b011……, and a header payload of 1 is used for true values, so the final value is 0b01100001, which is the above-mentioned 0x61. This process is repeated for the next key–value pair following the same encoding rules for wire-types and header payloads.

## 4. Evaluation Methodology

The evaluation methodology used in this research had two main parts: the design of the research and the realization of the tests to compare data serialization formats. In this section, the design of the research carried out to compare data serialization formats is described. Four main aspects were defined in order to obtain a useful comparison of the formats enumerated above:

1. Attributes;
2. Hardware;
3. Libraries;
4. Tests and Payloads.

### 4.1. Attributes

The attributes or characteristics measured to compare data serialization formats are the following:

1. Serialization/Deserialization speed. The values were measured per 1000 iterations and are expressed in microseconds.
2. Binary size increase with the use of the library. This attribute is very important with memory-limited devices such as Arduino UNO and is necessary for its application in IoT devices.
3. Encoding sizes. This attribute is very important when on a limited bandwidth network and, in consequence, for the scope of this study.

### 4.2. Hardware

To perform these tests, Arduino UNO (BCMI LABS LLC, Scarmagno, Italy) and ESP32-WROVER-B (Espressif Systems, Shanghai, China) modules were used. Due to their different characteristics, these devices represent different use scenarios. Arduino UNO represents a device with low memory capacity and low CPU power, while ESP32-WROVER-B represents a device with higher memory and higher CPU. The tests were performed on both devices to show the results in both cases.

#### Hardware Characteristics

Three key characteristics were involved in the tests:

- Flash memory, also called program space: where the compiled code is saved;
- SRAM: the memory where the variables and the dynamic code are loaded and read;
- CLK speed: the CPU clock speed.

Arduino UNO characteristics:

- Flash memory: 32 KB;
- SRAM: 2 KB;
- CLK speed: 16 MHz.
  Arduino UNO has very limited memory to store the program and the variables, so some of the performed tests only work on ESP32.

ESP32-WROVER-B characteristics:

- Flash memory: 1310 KB;
- SRAM: 327 KB;
- CLK speed: 240 MHz.

### 4.3. Libraries

This section contains the libraries used for each protocol. Some libraries are not supported on Arduino UNO because they have dependencies not available with it.

The libraries used for each protocol are:

- JSON: ArduinoJson. Library: https://arduinojson.org/ (accessed on: 19 February 2021). Version used: 6.18.0
- MessagePack: ArduinoJson. Library: https://arduinojson.org/ (accessed on: 19 February 2021). Version used: 6.18.0
- Protocol Buffers: NanoPB. Library: https://github.com/nanopb/nanopb (accessed on: 19 February 2021). Version used: 0.4.5
- Protoson: Pson. Library: https://github.com/thinger-io/Protoson (accessed on: 19 February 2021). Version used: https://github.com/thinger-io/Protoson/commit/60537257cb52a5a16ad2e5444226ebab82a7ceb1
- XML: TinyXML2. Library: https://github.com/leethomason/tinyxml2 (accessed on: 19 February 2021). Version used: 8.0.0
- BSON: MiniBSON. Library: https://github.com/cyberguijarro/minibson (accessed on: 19 February 2021). Version used: https://github.com/cyberguijarro/minibson/commit/3a460446245b17ffc3947f02a079b2232cef973a
- Avro: Apache Avro. This library was not used on the microcontrollers because there are currently no implementations for it. They were implemented in Java and executed on a computer in order to obtain the serialized object and measure its size for serialization and deserialization. For this reason, the tests with this library only contain data on sizes. The associated code can be found in the theoretical-tests folder.
- Thrift: Apache Thrift. This library was not used on the microcontrollers because there are currently no implementations for it. They were implemented in Java and executed on a computer in order to obtain the serialized object and measure its size for serialization and deserialization. For this reason, the tests with this library only contain data on sizes. The associated code can be found in the theoretical-tests folder.
- YAML. This library was not used on the microcontrollers because there are currently no implementations for it. They were implemented in Java and executed on a computer in order to obtain the serialized object and measure its size for serialization and deserialization. For this reason, the tests with this library only contain data on sizes. The associated code can be found in the theoretical-tests folder.

Table 2 describes the details of all the libraries used.

### 4.4. Tests and Payloads

Different tests and payloads were developed to measure each defined attribute. For each attribute, the payloads are the following:

1. Serialization/Deserialization speed. The tests used to measure this attribute were performed by using 10 different payloads and checking the time needed to serialize and deserialize them.
2. Binary size increase with the use of the library. The tests used to measure this attribute were performed using a reference code (code-without-library folder) to measure the binary size generated when not using any library. Then, the code from the binary-size-tests folder was loaded on each microcontroller, and the binary size increment was calculated.
3. Encoding sizes. This attribute is very important when on a limited bandwidth network and, in consequence, for the scope of this study. The tests used to measure this attribute were performed by using 10 different payloads and checking the generated serialized object size.

The 10 payloads used to measure the results for the encoding size and speed tests are named Test#. All of the tests performed are labeled with their name and can be checked here to confirm which payload was used for any test (shown as their JSON representation):

1. Test01
  
			{
			    "sensor":"gps",
				"time":1351824120,
				"data":[
				    48.75,
				    2.3
				]
		         }
2. Test02
  
			{
			    "sensor":"This is a very long string. This is a very
				long string. This is a very long string. This is a very
				long string. This is a very long string. ",
				"time":1351824120,
				"data":[
				  48.75,
				  2.3
				]
			}
3. Test03
  
			{
			    "sensor":"This contains a lot of keys.",
				"sensor2":"This contains a lot of keys.",
				"sensor3":"This contains a lot of keys.",
				"sensor4":"This contains a lot of keys.",
				"sensor5":"This contains a lot of keys.",
				"sensor6":"This contains a lot of keys.",
				"sensor7":"This contains a lot of keys.",
				"time":1351824120,
				"data":[
				  48.75,
				  2.30
				]
			}
4. Test04
  
			{
			    "data":[
				48.75,
				2.3,
				3.01,
				5.4,
				6.7,
				4.3,
				10.01,
				10.01
				]
			}
5. Test05
  
			{
			    "bool":true
			}
6. Test06
  
			{
			    "neg":-2
			}
7. Test07
  
			{
			    "pos":1
			}
8. Test08
  
			{
			    "double":1.03
			}
9. Test09
  
			{
			    "string":"test"
			}
10. Test10
  
			{
			    "string":"test",
				"double":1.03,
				"long":1351824120,
				"pos":1,
				"neg":-2,
				"bool":true,
				"array":[
				  48.75,
				  2.3
				]
			}

## 5. Research Results

This section summarizes the research results with new findings for each of the attributes studied.

### 5.1. Serialization/Deserialization Speed

These are the values measured for each test; the test definitions above can be referenced to confirm which payload corresponds to each row. As previously mentioned, the values were measured per 1000 iterations and are expressed in microseconds. The results are presented for ESP32-WROVER-B and Arduino UNO in two separate tables.

These tests show the performance of each library and protocol. High values for the serialization and deserialization time indicate that more CPU cycles were used for the data processing, which leads to more power consumption. Power consumption is very important in scenarios in which devices are powered by batteries. In addition, decreased use of the CPU by the serialization and deserialization process allows the device to use it for its actual goal, i.e., reading the sensors and processing their data. In these tests, the lower the values, the better.

**Figure 3:**
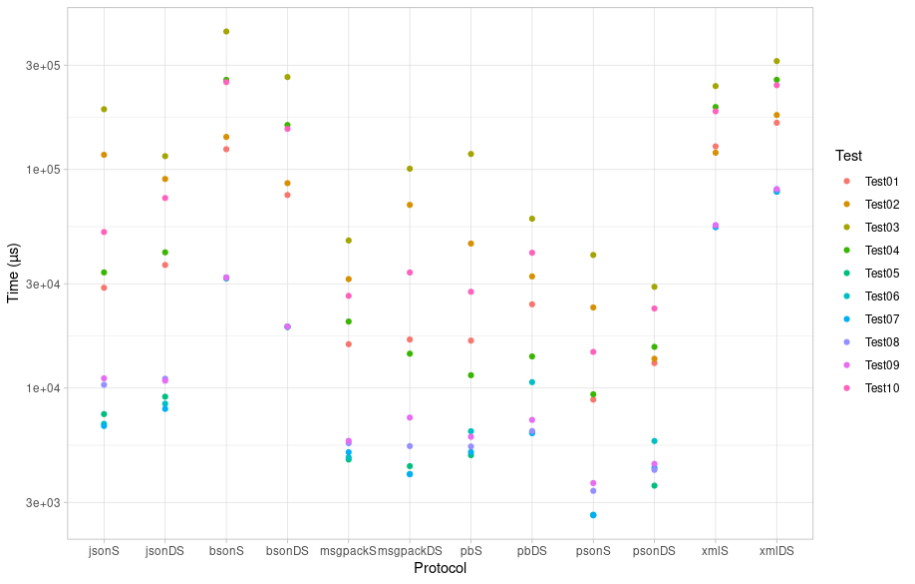
ESP32 Serialization (S)/Deserialization (DS) speed.

The results for ESP32 are reported in Figure 3 and Table 3.

**Figure 4:**
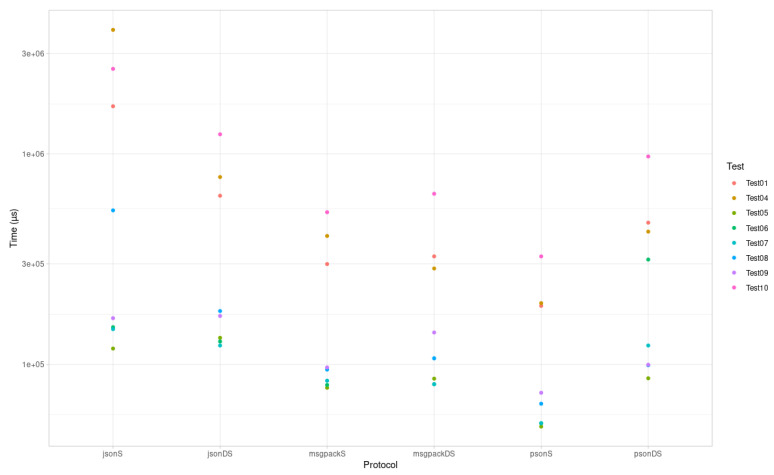
Arduino UNO Serialization (S)/Deserialization (DS) speed.

The results for Arduino are depicted in Figure 4 and Table 4.

### 5.2. Binary Size Tests

This section presents the results of the binary size tests for each format. These values are important when working with microcontrollers. Due to their limited memory, it is important to keep the utility code as small as possible. If the serialization/deserialization library consumes a lot of the total memory of the device, there may be insufficient memory to load the code that is necessary to perform the actual microcontroller task. In these tests, the lower the values, the better. The binary sizes without a library are:

- Arduino: 592 bytes;
- ESP32: 260,710 bytes.

The total available memory on each device is:

- Arduino: 32,256 bytes;
- ESP32: 1310,720 bytes.

**Figure 5:**
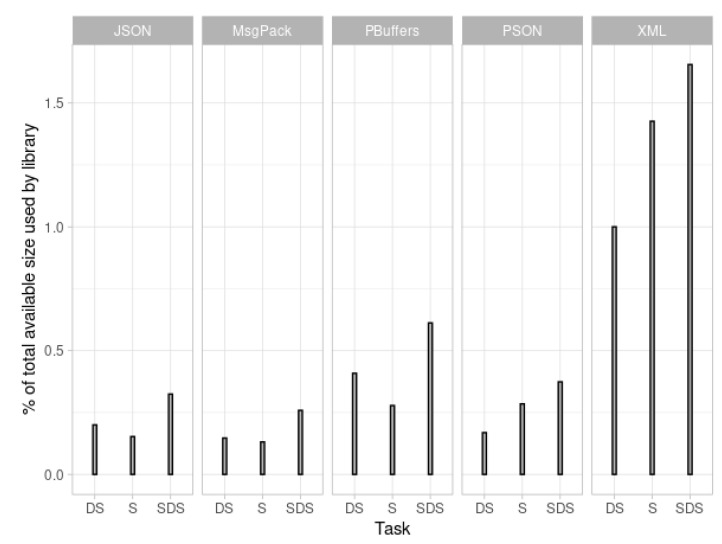
ESP32 binary size increase.

**Figure 6:**
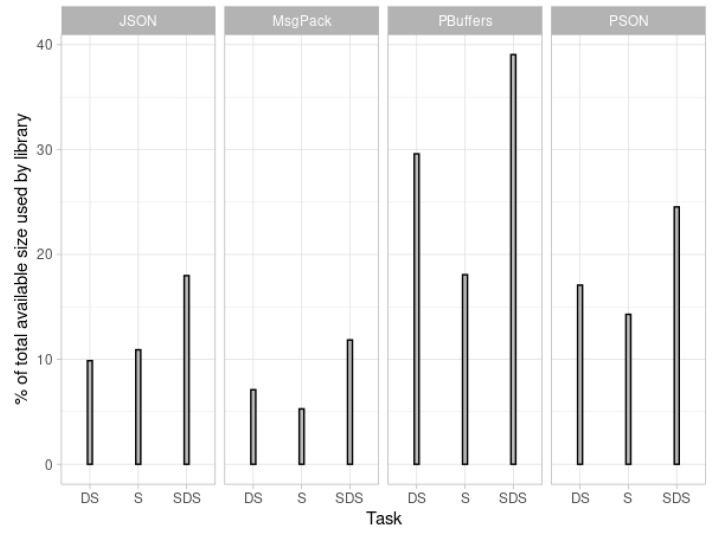
Arduino binary size increase.

The calculated percentages for the sizes are presented in Figure 5 and Figure 6. The “Increase” columns in the figures represent the actual percentage of program size increase resulting from the addition of the serialization and deserialization library (BSON is not represented in Figure 5 because its values are very different from the rest and would distort the whole graphic). This value is calculated by subtracting the binary size without a library from the total value obtained in the test.

### 5.3. Encoding Sizes

**Figure 7:**
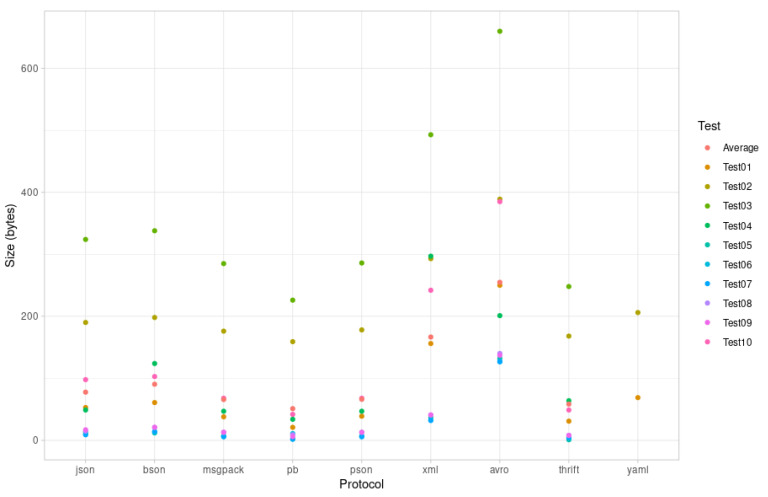
Protocol encoding sizes.

These are the results obtained after encoding each Test# payload with each library. These values are important because microcontrollers are commonly used in low-bandwidth or mesh networks. In these scenarios, sending a message through the net has a high cost in terms of network capacity. Furthermore, sending or receiving a larger message uses more of the network interface, so the device consumes more power. As described in the serialization/deserialization speed section, this is very important when devices are powered by batteries. In these tests, the lower the value, the better. Figure 7 includes the results for Avro, Thrift, and YAML, although they were not used in the microcontrollers. Avro, Thrift, and YAML messages were serialized using Java on a computer, and their serialized sizes were measured for each test. All results are shown in bytes.

Figure 7 shows the size values for each serialization protocol.

The above evaluation results for encoding time, encoding size, and binary size increase indicate that PSON is quite an efficient mechanism for embedded systems. According to the encoding time, PSON is one of the fastest formats for completing the encoding/decoding tests for ESP32 (Figure 3), followed by Protocol Buffers or MessagePack. Compared to MessagePack (another schema-less format, similar to PSON), PSON is much faster on average: 33% faster encoding and 55% faster decoding. On Arduino UNO, as shown in Figure 4, it encodes 40% faster on average, but deserialization is around 47% slower. Thus, PSON is quite efficient when encoding information on microcontrollers, which is the normal scenario in IoT, where devices send information periodically to the cloud. From the encoding size results in Figure 7, the most efficient means of encoding information in these tests was obtained with Protocol Buffers or Apache Thrift, but this is only suitable for use cases in which the message structure is known beforehand by both the microcontroller and the server decoding the messages. However, this is not the standard scenario in the IoT ecosystem, as it will require creating custom decoding functions in the cloud for every message sent by the device, which typically implies the compilation of the format definition, the use of the generated source files, etc. This is not practical or sustainable in the long term when dealing with multiple device types or changes in the protocol, which may result in versioning complexity. Moreover, it complicates the cloud inter-operability required in IoT with third-party services or applications, which usually work over the well-known REST API schema, using JSON as the standard encoding format. Among the other schema-less encoding formats that can be directly converted to JSON, PSON is one of the most efficient methods and is comparable to MessagePack, obtaining quite similar results for encoding size. On average, PSON and MessagePack generate payloads that are 15% smaller than a raw JSON, but depending on the payload, it can be improved by 30–40%, i.e., in Test01 and Test06. Finally, the increases in binary size due to serialization/deserialization in Figure 5 and Figure 6 illustrate that the ArduinoJSON library, which provides serialization/deserialization for JSON and MessagePack, is quite an optimized library in this respect, leading with a smaller program footprint in both ESP32 and Arduino UNO. The PSON library is quite similar to ESP32, with a 20.26% increase above the baseline binary versus the 20.15% required for MessagePack or 20.21% required for JSON. For Arduino UNO, PSON is less optimized and results in a 10% greater footprint on average against Arduino JSON. In sum, PSON is a new encoding format that competes with MessagePack in terms of encoding size but improves the serialization time by using a simpler encoding approach. It outperforms other schema-less encoding systems such as JSON, XML, and BSON in both serialization size and time. The results also indicate that the PSON library competes with other specific state-of-the-art Arduino Libraries on modern microcontrollers such as ESP32, but it can be improved on more modest architectures such as the AVR used in Arduino.

## 6. Conclusions and Future Work

We are witnessing the emergence of a new generation of IoT devices capable of being part of massively scalable and cost-effective IoT applications using LPWAN and the latest NB-IoT and LTE-M communication technologies. The deployment of massive IoT applications requires an huge volume of low-cost, low-power sensor devices. Therefore, these sensor devices will have relatively low performance requirements.

In an IoT environment, clients and servers exchange data. Part of this data may be in the transport protocol, which is used in the exchange of messages. Within messages, structured information (integers of different sizes and formats, arrays, strings, etc.) can be exchanged. Therefore, the use of serialization formats is necessary to represent this structured data in a linear set of bytes, which can be equivalently deserialized. The process of serialization and deserialization is critical in massive IoT environments, as it consumes processing time and has an impact on message size, and consequently, it is directly related to the energy consumption of sensor devices and their lifetime. This paper presents a new serialization format used by the Thinger.io platform called PSON. PSON optimizes execution time, channel utilization, and power consumption compared to the most common methods used in IoT environments.

In order to evaluate the efficiency of PSON, tests were carried out to compare it with the most widely used serialization formats using different payloads. The evaluation results demonstrate the excellent performance of PSON in terms of serialization, deserialization, and average encoding sizes. Specifically, the serialization and deserialization times for 1000 iterations were 11,179 μs and 11,657 μs for ESP32 and 125,671 μs and 324,406 μs for Arduino UNO; the average encoding binary size was 66.3 bytes. PSON also presented good results for library binary size overhead.

Future works will involve extending this research to other IoT services, such as the performance impact in mesh and low-bandwidth networks and the energy savings for microcontrollers. Moreover, the intention is to register the encoding format within IANA (Internet Assigned Numbers Authority) so that PSON can become a new standard in the future for optimizing JSON payloads over the Internet. There is also an opportunity to improve PSON libraries by reducing the compile size in microcontrollers, thus increasing the efficiency in constrained devices. In addition, there is a plan to create libraries in different languages, such as Python, Node.JS, and Java, so the encoding format can be much more operable with different programming languages and custom back-ends. Finally, the improvement of libraries is planned with the use of zero-copy techniques [42] to avoid unnecessary memory copying to improve deserialization time. 

## Figures and Tables

**Table 1:** Encoding rules for different PSON wire types.

Header			Value
Wire Type	Header Payload	Binary Representation	
Unsigned	Unsigned integer up to 0x1E or 0x1F to signal an upcoming varint.	0 0 0 [P P P P P]	LEB128 if integer is greater than 0x1E
Signed	Signed integer up to 0x1E or 0x1F to signal an upcoming varint.	0 0 1 [P P P P P]	LEB128 if integer is greater than 0x1E
Floating Point	0x00 to indicate that the floating point is single precision.	0 1 0 [0 0 0 0 0]	Stores a floating point number in IEEE 754 single-precision floating point number (fixed to 32 bits)
Floating Point	0x01 to indicate that the floating point is double precision.	0 1 0 [0 0 0 0 1]	Stores a floating point number in IEEE 754 double-precision floating point number (fixed to 64 bits)
Discrete	0x00 to indicate False	0 1 1 [0 0 0 0 0]	N/A
Discrete	0x01 to indicate True	0 1 1 [0 0 0 0 1]	N/A
Discrete	0x02 to indicate Null	0 1 1 [0 0 0 1 0]	N/A
String	Unsigned integer up to 0x1E to indicate the string size, or 0x1F to signal an upcoming varint to specify the size.	1 0 0 [P P P P P]	LEB128 if string length is greater than 0x1E
			UTF-8 String
Bytes	Unsigned integer up to 0x1E to indicate the byte array size, or 0x1F to signal an upcoming varint to specify the size.	1 0 1 [P P P P P]	LEB128 if byte array length is greater than or equal to 0x1E
			Binary data
Map	Unsigned integer up to 0x1E to indicate the number of elements present in the map, or 0x1F to signal an upcoming varint to specify the size.	1 1 0 [P P P P P]	LEB128 if byte array length is greater than 0x1E
			Map Data
Array	Unsigned integer up to 0x1E to indicate the number of elements present in the array, or 0x1F to signal an upcoming varint to specify the size.	1 1 1 [P P P P P]	LEB128 if byte array length is greater than 0x1E
			Array Data

**Table 2:** Libraries used for comparison of data serialization protocols.

Protocol	Format	Library	Header only	Static Memory	Library Size (Not Compiled)
JSON	JSON	ArduinoJSON	yes	yes	227,185 bytes
MsgPack	MsgPack	ArduinoJSON	yes	yes	227,185 bytes
ProtocolBuffers	ProtocolBuffers	Nano PB	no	yes	42,000 bytes
Protoson	Protoson	Protoson	yes	yes	31,294 bytes
XML	XML	TinyXML2	yes	yes	151,373 bytes
BSON	BSON	MiniBSON	yes	yes	18,152 bytes
Apache Avro	This library was not used on the microcontrollers and was tested in Java				
Apache Thrift	This library was not used on the microcontrollers and was tested in Java				
YAML	This library was not used on the microcontrollers and was tested in Java				

**Table 3:** ESP32 Serialization (S)/Deserialization (DS) speed.

Protocol	Test01	Test02	Test03	Test04	Test05	Test06	Test07	Test08	Test09	Test10	Average
JSON S	28,767	116,705	188,648	33,814	7602	6865	6708	10,350	11,082	51,759	46,230
JSON DS	36,577	90,513	115,070	41,774	9132	8481	8033	11,032	10,809	74,032	40,545.3
BSON S	123,806	140,927	427,762	256,979	31,812	31,846	31,837	32,081	32,020	250,959	136,002.9
BSON DS	76,403	86,572	264,767	159,726	19,020	19,092	19,083	19,114	19,090	153,429	83,629.6
MsgPack S	15,875	31,524	47,345	20,153	4714	4819	5085	5597	5737	26,417	16,726.6
MsgPack DS	16,677	68,870	100,840	14,366	4394	4047	4034	5427	7330	33,787	25,977.2
Protocol Buffers S	16,487	45,835	117,739	11,449	4931	6347	5093	5403	5986	27,574	24,684.4
Protocol Buffers DS	24,162	32,412	59,527	13,952	6286	10,642	6216	6360	7152	41,606	20,831.5
PSON S	8854	23,372	40,624	9364	2627	2632	2617	3387	3676	14,637	11,179
PSON DS	13,011	13,618	29,044	15,432	3578	5726	4318	4225	4500	23,119	11,657.1
XML S	127,529	119,323	240,721	193,044	55,073	54,538	54,506	55,618	55,504	184,497	114,035.3
XML S	163,530	177,467	313,182	257,228	80,087	79,212	79,159	81,217	81,216	242,851	155,514.9

**Table 4:** Arduino UNO Serialization (S)/Deserialization (DS) speed.

Protocol	Test01	Test02	Test03	Test04	Test05	Test06	Test07	Test08	Test09	Test10	Average
JSON S	1,685,840	-	-	3,890,896	118,868	150,172	147,008	539,372	165,756	2,539,444	1,154,669.5
JSON DS	633,272	-	-	776,852	133,652	128,432	122,884	179,188	169,768	1,240,716	423,095.5
BSON S	-	-	-	-	-	-	-	-	-	-	-
BSON DS	-	-	-	-	-	-	-	-	-	-	-
MsgPack S	299,712	-	-	407,748	77,536	79,980	83,780	94,592	96,824	528,548	208,590
MsgPack DS	325,988	-	-	285,564	85,656	80,604	80,632	106,908	141,716	647,048	219,264.5
Protocol Buffers S	-	-	-	-	-	-	-	-	-	-	-
Protocol Buffers DS	-	-	-	-	-	-	-	-	-	-	-
PSON S	189,648	-	-	195,420	50,688	52,536	52,632	65,096	73,408	325,940	125,671
PSON DS	471,604	-	-	427,496	86,000	314,908	122,880	99,216	99,776	973,368	324,406
XML S	-	-	-	-	-	-	-	-	-	-	-
XML S	-	-	-	-	-	-	-	-	-	-	-

## Data Availability

Not applicable.
